# Differences in macular thickness associated with peripheral retinal vessel whitening in diabetic patients

**DOI:** 10.1038/s41598-024-68839-0

**Published:** 2024-08-27

**Authors:** Fritz Gerald P. Kalaw, Paripoorna Sharma, Evan Walker, Shyamanga Borooah

**Affiliations:** 1 Jacobs Retina Center, University of California San Diego, La Jolla, CA 92093 USA; 2 The Viterbi Family Department of Ophthalmology and Shiley Eye Institute, University of California San Diego, 9415 Campus Point Dr, La Jolla, CA 92093 USA; 3 Division of Ophthalmology Informatics and Data Science, The Viterbi Family Department of Ophthalmology and Shiley Eye Institute, University of California San Diego, La Jolla, CA 92093 USA

**Keywords:** Eye diseases, Diabetes complications

## Abstract

This study aimed to determine the difference in macular thickness among patients with diabetes mellitus (DM) with and without peripheral retinal vessel whitening (PRVW). PRVW was defined by retinal vessel whitening outside the standard seven ETDRS fields. Subjects were divided into DM with PRVW, DM without PRVW, and normal age-matched controls. Optical coherence tomography scans were divided into total, inner, and outer retinal layer thicknesses and were compared in the macula's central, inner, and outer rings. Forty-seven eyes were included: DM with PRVW = 15, DM without PRVW = 16, and Controls = 16. Overall, the mean retinal thickness in patients with DM with PRVW was lower than in patients with DM without PRVW and controls. In the inner macula, DM patients with PRVW showed a significantly lower mean inner superior, nasal, inferior, and temporal macula compared to DM patients without PRVW (*p* = 0.014, 0.008, 0.005, < 0.001, respectively). DM patients with PRVW also showed a significantly lower mean outer superior, nasal, inferior, and temporal macula than controls (*p* = 0.005, 0.005, 0.016, 0.025, respectively). This study demonstrates that PRVW in DM patients may be associated with global structural changes to the macular region, promoting a decrease in inner and outer retinal thickness. Further studies should investigate the functional correlation with PRVW in DM patients in order to better understand its potential implications in diabetic patients.

## Introduction

In 2021, 537 million adults 20–79 years of age were estimated to have diabetes mellitus (DM)^1^. In the United States (US) alone, DM affects 38.4 million people (11.6% of the US population) and is the leading cause of new cases of blindness among adults 18–64 years of age^2^. One quarter (9.60 million people), among these DM cases, were estimated to have diabetic retinopathy (DR), 1.84 million of which had vision-threatening DR^3^.

A proposed clinical classification system (Diabetic Retinopathy Disease Severity Scale [DRSS]) based on retinal manifestations was introduced to improve the screening and management of DR in DM patients. These clinical findings consist of the following: microaneurysms, intraretinal hemorrhages, venous beading, intraretinal microvascular abnormalities, neovascularization, or vitreous/preretinal hemorrhages. Depending on the findings observed on dilated ophthalmoscopy, the DR severity varies from no apparent retinopathy to non-proliferative diabetic retinopathy (NPDR) in mild, moderate, and severe stages, to the more advanced cases, which include proliferative diabetic retinopathy (PDR)^4^. As DR severity increases, the retina exhibits vision-threatening manifestations, with diabetic macular edema (DME) being the most common cause of vision reduction^5,6^. DME is mediated mainly by vascular endothelial growth factors (VEGF) and dysregulated growth factor levels, which cause hyperpermeability of the retinal blood vessels and capillaries, eventually leading to retinal thickening or hard exudates in the posterior pole^4–6^ Other vision-threatening causes of DR are tractional retinal detachment, vitreous hemorrhage, and neovascular glaucoma^6^. Additionally, in ischemic retinal diseases, hypoxic processes are characterized by arteriolar or venular retinal vessel whitening due to the accumulation of material and resultant blood flow deprivation. In our recent study^7^, in a cohort of patients with DM, we found that the presence of peripheral retinal vessel whitening (PRVW), defined as the presence of whitening of retinal arteriole or venule outside the standard seven Early Treatment Diabetic Retinopathy Study (ETDRS) fields, suggested an association with increasing DRSS. In addition, PRVW was associated with a reduced best-corrected visual acuity. The reason for this association was not clear.

Despite the normal-appearing cross-sectional anatomy of the neurosensory retina, our group hypothesized that the vision disparity resulted from structural changes in the macula. This study aims to determine the difference in macular thickness measurements among DM patients, stratifying DM patients with and without PRVW.

## Methods

### Study design and participants

This retrospective, cross-sectional study was approved by the University of California San Diego (UCSD) Institutional Review Board and followed the guidelines of the Helsinki Declaration. Informed consent was obtained per the institution protocol, and data collection and analysis were anonymized and complied with the Health Insurance Portability and Accountability Act (HIPAA) of 1996.

The subjects included consecutive DM patients seen by retinal specialists at the Shiley Eye Institute, UCSD, between September 2020 and December 2021. These subjects were confirmed DM patients who visited the eye clinic for either DR screening or established cases of DR undergoing monitoring. The subjects underwent routine comprehensive ophthalmologic examination and were subsequently diagnosed by the retinal specialist as having no apparent retinopathy, mild NPDR, moderative NPDR, severe NPDR, or PDR. In addition to the ophthalmologic examination, patients underwent multi-modal retinal imaging, including ultra-wide field imaging and spectral-domain optical coherence tomography (SD-OCT) as standard for clinic protocol. Patients with PRVW and without PRVW outside the seven standard ETDRS fields were separated. Images captured from ultra-wide field system (Optos plc, Dunfermline, UK) were processed from the imaging database and the seven standard ETDRS fields were manually interpolated for each image using Microsoft Powerpoint (version 16.58, Microsoft Corporation, Redmond, WA). Two experienced ophthalmologists independently identified the retinal vessel whitening outside the ETDRS fields. In cases of disagreement, a repeat review and consensual discussion of the images were performed. Additionally, age-matched controls were obtained from consecutive patients who presented to the eye clinic between January 2019 and December 2020 for routine eye examination and were confirmed by a retinal specialist not to have retinal pathology nor PRVW, as well as any systemic disease. Both eyes of subjects were included, if eligible.

Patients were excluded if one or more of the following were noted: media opacity obscuring visualization of the retina on imaging, prior retinal laser procedure (focal laser or panretinal photocoagulation), or other history or concomitant active retinal pathologies (retinal detachment, age-related macular degeneration, central or branch retinal occlusion, macular edema, pathologic myopia). Prior laser procedures and retinal occlusions were excluded since these cases could induce whitening of retinal vessels. Any eyes with macular pathology were also excluded to avoid confounding disease for macular thickness measurements.

### Structural SD-OCT image protocol and analysis

SD-OCT scans were captured using Heidelberg HRA + OCT Spectralis System (Heidelberg Engineering, Heidelberg, Germany). The following pattern was obtained on each de-identified patient for image analysis: number of B-scans—49, pattern size—20° × 20° (6.1 × 6.1 mm), distance between scans—127 μm, with automatic real-time tracking enabled.

Macular thickness measurements, centered on the fovea, were measured and analyzed using the built-in proprietary Heidelberg HRA + OCT Spectralis software (viewing module v7.05.0). The retinal thickness map provided the average retinal thickness measurements of the following sectors: central subfield (1 mm), inner superior/nasal/inferior/temporal (2 mm), and outer superior/nasal/inferior/temporal (3 mm).

**Figure 1:**
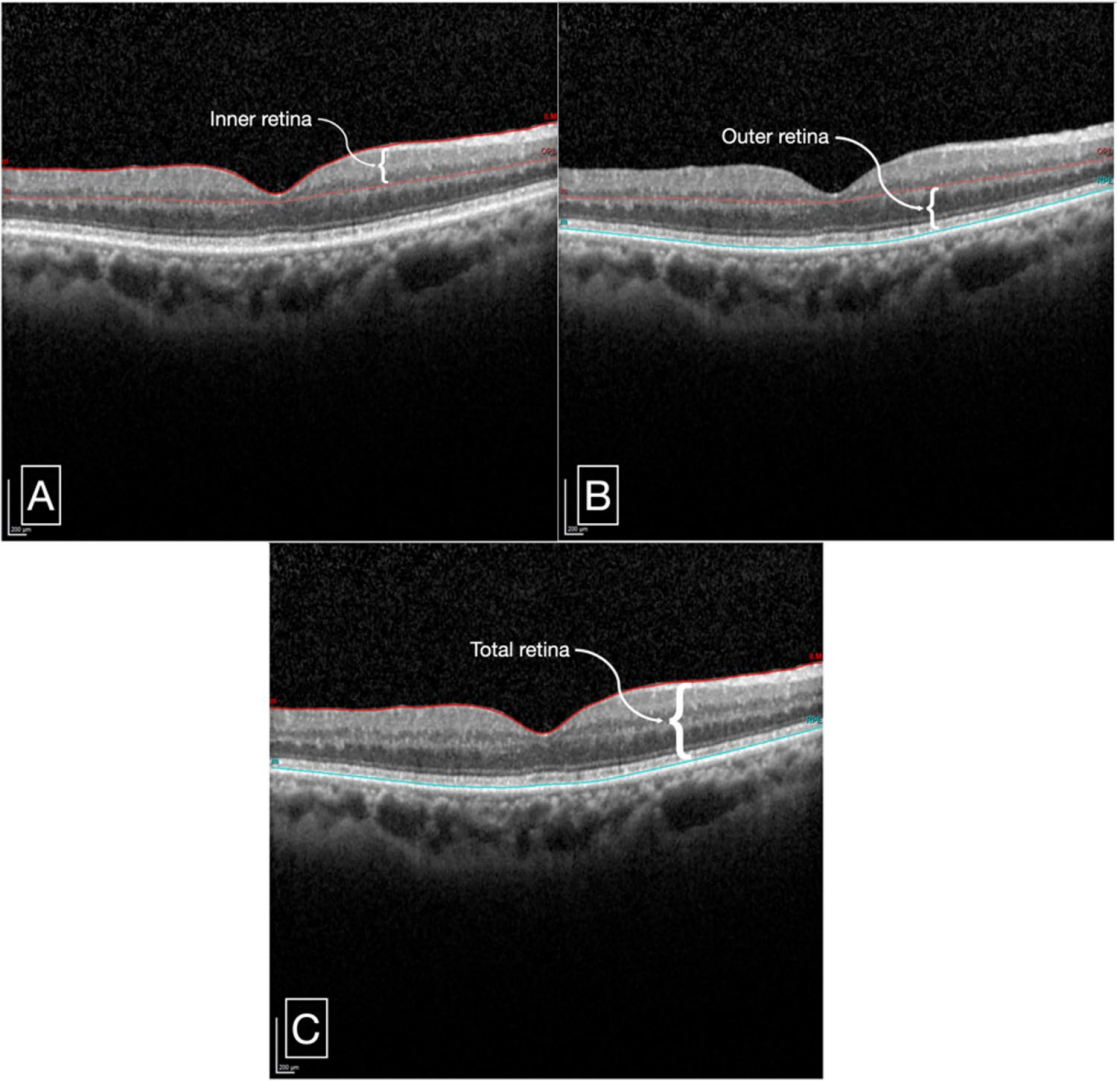
A representative cross-sectional SD-OCT scan of a patient with DM with PRVW showing retinal thickness measurement segmentation of the following layers: Inner retinal thickness—distance between ILM and the inner border of the OPL (**A**), Outer retinal thickness—distance between the inner border of the OPL and the inner border of the RPE (**B**), and total retinal thickness—distance between ILM and inner border of RPE (**C**). *SD-OCT* spectral-domain optical coherence tomography, *ILM* internal limiting membrane, *OPL* outer plexiform layer, *RPE* retinal pigment epithelium.

A meticulous semi-automated segmentation was performed by one of the masked image analyzers. Retinal thickness measurements were divided into three parameters (Fig. 1):

- *Inner retinal thickness* the distance between the internal limiting membrane (ILM) and the inner border of the outer plexiform layer (OPL).
- *Outer retinal thickness* the distance between the inner border of the OPL and the inner border of the retinal pigment epithelium (RPE).
- *Total retinal thickness* the distance between the ILM and the inner border of the RPE, or the sum of the inner retinal and outer retinal thicknesses.

### Statistical analysis

Demographic and clinical parameters were compared between cohorts using Fisher’s Exact Test and Analysis of Variance (ANOVA) for patient-level categorical and continuous variables, respectively. Mean retinal sectoral thickness measurements were estimated per cohort utilizing linear mixed-effects models, with random intercepts to account for within-subject variability to adjust for the correlation between two eyes being included from the same patient. A post-hoc analysis for pairwise comparisons between cohort mean thickness measurements was carried out using Tukey’s Test with degrees of freedom estimated by Satterthwaite’s approximation. All mean retinal thickness estimates presented were derived using models that adjusted for patient age. Age adjustments were incorporated due to its confounding relationship with retinal thickness, ensuring thickness estimates were accurately compared between groups independent of age-related variations. Statistical analysis was performed using R software. P-values ≤ 0.05 were assigned significance.

## Results

The study included 47 eyes from 34 subjects. Fifteen eyes from 13 patients with DM with PRVW, 16 eyes from 13 patients with DM without PRVW, and bilateral eyes of 8 age-matched control subjects were included. The age, sex, race, and ethnicity were not statistically significantly different between the three cohorts. (Table 1) The distribution of DR diagnoses across the three cohorts is shown in Table 2.

**Table 1:** Demographic characteristics of patients across cohorts.

	DM with PRVW	DM without PRVW	Control	p-value
Age	62.1 (55.4, 68.8)	56.2 (43.6, 68.8)	70.3 (65.2, 75.3)	0.130
Sex				
Female	7 (53.8%)	5 (38.5%)	4 (50.0%)	0.757
Male	6 (46.2%)	8 (61.5%)	4 (50.0%)	
Race				
Asian	2 (15.4%)	5 (38.5%)	0 (0.0%)	0.235
Black	2 (15.4%)	2 (15.4%)	0 (0.0%)	
Native Hawaiian or Pacific Islander	0 (0.0%)	1 (7.7%)	0 (0.0%)	
Unknown	2 (15.4%)	2 (15.4%)	2 (25.0%)	
White	7 (53.8%)	3 (23.1%)	6 (75.0%)	
Ethnicity				
Hispanic	3 (23.1%)	1 (7.7%)	1 (12.5%)	0.825
Non-Hispanic	10 (76.9%)	12 (92.3%)	7 (87.5%)	

*DM* diabetes mellitus, *PRVW* peripheral retinal vessel whitening.

**Table 2:** Distribution of DR diagnoses of patients’ eyes across three cohorts.

DR severity score	DM with PRVW	DM without PRVW	Controls*	Total
No retinopathy	7	8	16	31
Mild NPDR	4	4	0	8
Moderate NPDR	0	0	0	0
Severe NPDR	0	0	0	0
PDR	4	4	0	8
Total	15	16	16	47

*DR* diabetic retinopathy, *DM* diabetes mellitus, *NPDR* non-proliferative diabetic retinopathy, *PDR* proliferative diabetic retinopathy.

*Control subjects were patients included in the study who visited the eye clinic for routine eye examination and had no history or active retinal pathology nor systemic disease (i.e., DM).

Analysis of age-adjusted retinal thickness showed that, in general, the mean retinal thickness in subjects in the DM with PRVW group was lower than that of those with DM without PRVW and in controls. The overall mean total, inner, and outer retinal thickness measurements were statistically significantly lower in subjects with DM with PRVW in the inner 2 mm of the macula compared to subjects with DM without PRVW and controls. In subjects with DM without PRVW, the mean retinal thickness measurements were higher in the inner and lower in the outer retina than in controls, although this was not statistically significant. (Tables 3, 4, and 5) This suggests that the inner 2 mm of the macula was more significantly affected by PRVW in patients with DM.

**Table 3:** Total retinal thickness comparisons per ETDRS grid across cohorts.

Total retina	DM with PRVW	DM without PRVW	Control	DM with PRVW vs. without PRVW	DM with PRVW vs. Control	DM without PRVW vs. Control
Central	233.00 (210.33, 255.98)	240.17 (217.04, 263.00)	241.52 (193.96, 289.38)	0.266	0.478	0.910
Outer superior	281.47 (265.12, 297.77)	292.45 (275.83, 309.05)	295.65 (260.69, 330.62)	0.128	0.108	0.716
Outer nasal	268.14 (246.81, 289.43)	275.17 (253.44, 296.87)	278.26 (232.40, 324.27)	0.456	0.372	0.788
Outer inferior	292.37 (275.33, 309.41)	293.32 (275.69, 310.96)	298.28 (261.07, 335.49)	0.913	0.511	0.593
Outer temporal	276.42 (264.73, 288.21)	284.87 (272.67, 296.97)	290.89 (265.81, 317.08)	0.161	**0.026**	0.352
Inner superior	316.20 (295.47, 336.91)	337.89 (316.63, 359.17)	338.43 (293.09, 384.11)	**0.035**	**0.049**	0.962
Inner nasal	308.26 (289.48, 327.20)	332.92 (313.42, 352.46)	325.27 (284.51, 366.87)	**0.013**	0.097	0.461
Inner inferior	316.07 (301.51, 330.62)	336.96 (321.90, 352.02)	340.00 (308.22, 371.78)	**0.007**	**0.003**	0.701
Inner temporal	309.00 (293.19, 324.86)	324.85 (308.80, 340.91)	338.23 (304.77, 371.59)	**0.018**	**0.002**	0.125

*DM* diabetes mellitus, *PRVW* peripheral retinal vessel whitening.

*Bold p-values denote statistically significant values at less than 0.05.

**Table 4:** Inner retinal thickness comparisons per ETDRS grid across cohorts.

Inner retina	DM with PRVW	DM without PRVW	Control	DM with PRVW vs. without PRVW	DM with PRVW vs. Control	DM without PRVW vs. Control
Central	60.04 (46.21, 73.99)	59.32 (45.28, 73.24)	54.77 (25.77, 83.92)	0.854	0.472	0.537
Outer superior	137.41 (126.29, 148.51)	145.85 (134.57, 157.11)	144.32 (120.82, 167.74)	0.064	0.244	0.797
Outer nasal	154.83 (140.8, 168.81)	162.25 (147.96, 176.52)	160.73 (130.68, 190.81)	0.238	0.427	0.840
Outer inferior	157.21 (145.38, 169.01)	157.29 (145.20, 169.38)	158.41 (132.66, 184.29)	0.988	0.847	0.862
Outer temporal	138.29 (131.03, 145.55)	143.01 (135.50, 150.53)	145.39 (129.54, 161.24)	0.205	0.068	0.548
Inner superior	156.74 (145.28, 168.17)	171.30 (159.53, 183.06)	170.35 (144.88, 196.12)	**0.014**	**0.031**	0.879
Inner nasal	148.85 (136.91, 160.88)	163.50 (151.19, 175.70)	156.26 (130.76, 181.76)	**0.008**	0.251	0.269
Inner inferior	162.99 (153.08, 172.90)	177.93 (167.68, 188.19)	175.60 (153.96, 197.24)	**0.005**	**0.019**	0.665
Inner temporal	141.35 (133.46, 149.30)	157.17 (149.11, 165.25)	156.46 (139.31, 174.01)	** < 0.001**	**0.001**	0.867

*DM* diabetes mellitus, *PRVW* peripheral retinal vessel whitening.

*Bold p-values denote statistically significant values at less than 0.05.

**Table 5:** Outer retinal thickness comparisons per ETDRS grid across cohorts.

Outer retina	DM with PRVW	DM without PRVW	Control	DM with PRVW vs. without PRVW	DM with PRVW vs. Control	DM without PRVW vs. Control
Central	171.61 (160.51, 182.90)	180.81 (169.41, 192.02)	186.19 (163.06, 209.46)	**0.019**	**0.018**	0.365
Outer superior	141.80 (132.81, 150.79)	144.58 (135.44, 153.70)	149.23 (130.20, 168.21)	0.454	0.127	0.341
Outer nasal	140.30 (131.62, 149.02)	143.64 (134.69, 152.53)	147.76 (129.05, 166.52)	0.409	0.117	0.387
Outer inferior	138.99 (129.66, 148.32)	138.47 (128.81, 148.13)	143.04 (122.67, 163.42)	0.913	0.410	0.370
Outer temporal	135.05 (127.12, 142.97)	139.31 (131.27, 147.35)	142.81 (126.01, 159.56)	0.194	0.072	0.413
Inner superior	157.04 (147.91, 166.18)	164.33 (155.06, 173.58)	171.64 (152.39, 190.86)	**0.050**	**0.005**	0.143
Inner nasal	157.09 (147.33, 166.95)	166.00 (156.00, 175.93)	173.05 (152.36, 193.72)	**0.037**	**0.005**	0.188
Inner inferior	148.73 (140.19, 157.32)	155.88 (147.14, 164.61)	160.36 (142.09, 178.64)	0.072	**0.016**	0.340
Inner temporal	157.26 (148.95, 165.66)	161.61 (153.14, 170.00)	167.53 (150.14, 184.96)	0.127	**0.025**	0.187

*DM* diabetes mellitus, *PRVW* peripheral retinal vessel whitening.

*Bold p-values denote statistically significant values at less than 0.05.

**Figure 2:**
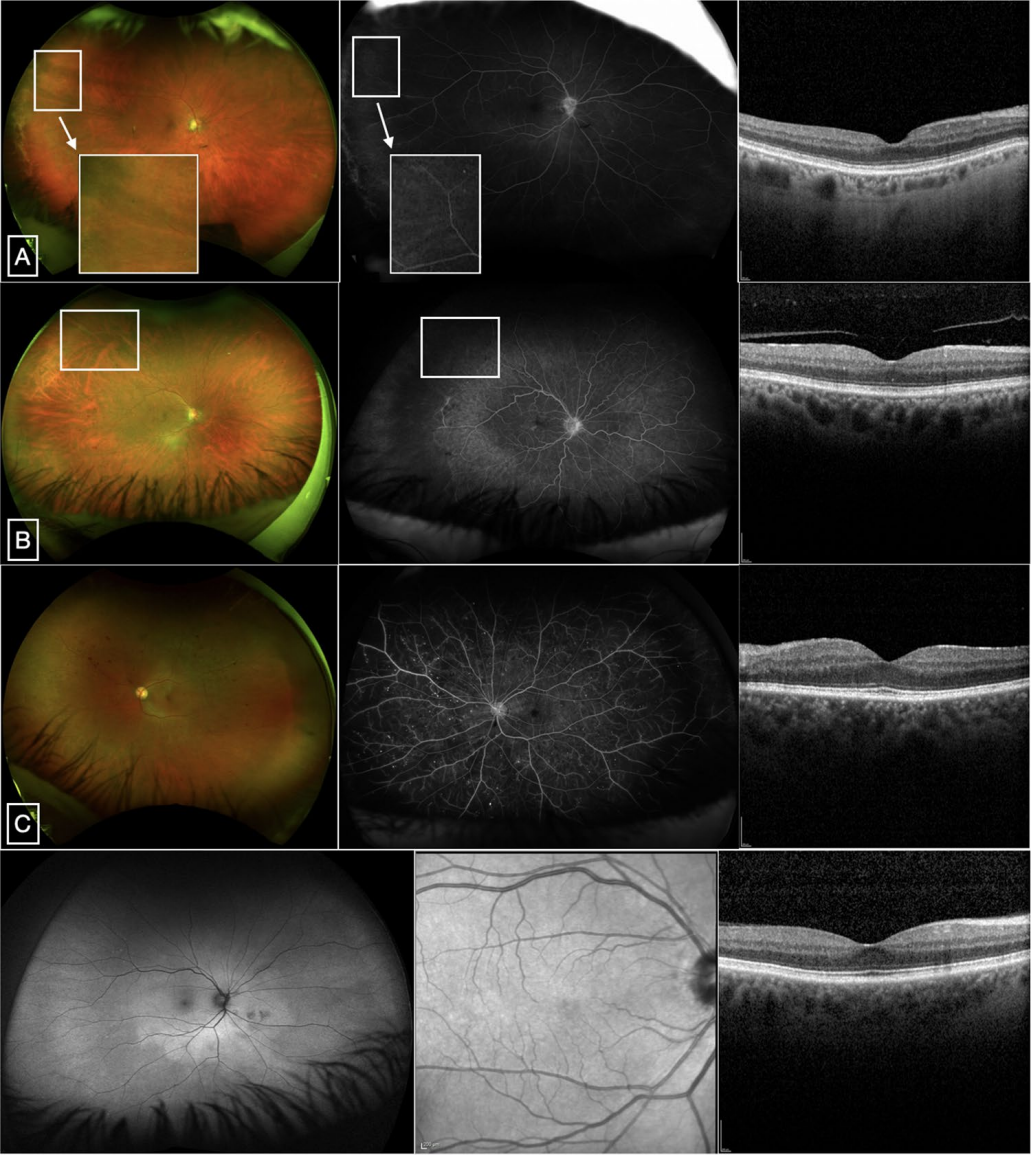
Representative multi-modal retinal images of DM patients included in the cohort. UWF pseudocolor (left), late-phase UWF FFA (middle), and SD-OCT (right) scans of patients with mild NPDR with PRVW (**A**,**B**) and PDR without PRVW (**C**). (**A**) Shows retinal vascular filling defect at the superotemporal periphery where PRVW is noted (box). Inset from the pseudocolor and FFA images show PRVW with corresponding vascular non-perfusion. (**B**) Shows vascular filling defect and capillary nonperfusion at the superotemporal periphery where PRVW is noted (box). (**C**) Shows multiple punctate hyperfluorescence with complete vascular filling and capillary perfusion. The area of the macula appears perfused without signs of ischemia in all subjects. *UWF* ultra-wide field, *FFA* fundus fluorescein angiography, *SD-OCT* spectral-domain optical coherence tomography, *NPDR* non-proliferative diabetic retinopathy, *PDR* proliferative diabetic retinopathy, *PRVW* peripheral retinal vessel whitening.

To first understand whether the vessels with retinal whitening were perfused and affecting macrovascular supply to the retina, we reviewed cases where fundus fluorescein angiography (FFA) was performed. We did not see vessel perfusion in the whitened vessels. As optical coherence tomographic angiography is not performed routinely in the clinic, we also examined the FFAs for signs of macular microvascular disease that could account for the thinning that was found in the DM patients with PRVW. Capillary nonperfusion, vascular filling defect, and signs of macular ischemia were not found (Fig. 2).

## Discussion

Much has been written about microvascular complications in DM, as described and classified in the DRSS. Aside from vascular involvement, DM also impairs the retinal neurovascular system^8^ and neural tissues, such as the neurosensory retina^9^. This study, which analyzed cross-sectional retinal images, revealed neurosensory alterations in patients with DM and discovered the following key findings: 1. Overall, the macular thickness was lower in DM eyes with PRVW than in DM eyes without PRVW and age-matched control eyes, with a significant difference in the inner 2 mm of the macula, 2. Inner retinal thickness was greater in DM eyes without PRVW than in age-matched control eyes, and 3. Outer retinal thickness was also greater in controls than in DM without PRVW.

The neurosensory retina possesses a dual circulation: the central retinal artery supplying the inner retina, and the choroid, particularly the choriocapillaris, indirectly supplying the avascular outer retina^10^. The retinal microvasculature is the primary gas and nutrient exchange site between blood and retinal tissues. In a similar manner to cerebral and peripheral nerve circulation, a disturbance with the microvasculature would compromise the nerve function^11^. This compromise of function may also, if severe enough, lead to the loss of retinal cells and, hence, retinal thinning. An interesting finding was that the thinning was found in both the inner and outer retina. This may be a result of neuroretinal and vascular remodeling^10^. Our group focused on identifying structural changes in DM patients based on the previous finding^7^ of visual acuity differences in those with PRVW.

The inner 2 mm of the retina comprises the fovea and parafovea. Since the fovea is avascular, we hypothesize that microvascular changes are less likely to affect this region. Also, as this region is so thin, a much larger study would be required to find potential significant differences. In the parafovea, the microvascular circulation spans from the ganglion cell layer to the inner nuclear layer^12^. In the present study, a significant difference in the inner 2 mm of the retina was noted, particularly in eyes with DM with PRVW. There were statistically significantly lower total retinal thickness measurements in patients with DM with PRVW compared to those without PRVW. When further divided into inner and outer retinal thickness, the inner 2 mm of the retina still demonstrated significant thinning in eyes with DM with PRVW. This decrease in retinal thickness measurements in the inner 2 mm of the retina was only statistically significantly different with the presence of PRVW since comparing DM patients without PRVW did not exemplify statistical significance when compared with age-matched controls. However, the inner retinal thickness of the inner 2 mm of the retina was higher in age-matched controls compared with DM patients without PRVW, although this was not statistically significant. The vulnerability of the inner 2 mm of the macula to ischemia and subsequent significant inner retinal thinning may be due to the insult within the arteriole, venule, and capillary beds, more evident in the parafoveal region. This could also be why the foveal avascular zone in DM patients is enlarged^13,14^. We did not find complete dropout in the macula in the DM eyes with PRVW who had FFAs. However, this does not exclude changes or reduction of the retinal vascular circulation or perhaps loss of a specific vascular plexus, such as the deep capillary plexus. Optical coherence tomography with angiography (OCTA) could better address whether there was microvascular loss. Unfortunately, as OCTA was not part of the routine clinic workup, these images were not available for the current study. Still, an association between macular microvascular changes in OCTA and PRVW would be interesting to study in future studies. The choroid, which supplies the outer retina, can also be affected by chronic hyperglycemia. Several studies analyzing choroidal morphology and vasculature have noted that choroidal thickness measurements are lower in patients with DM and DR when compared with controls^15–17^. Although not measured in the present study, this may be a cause for the lower outer retinal thickness in both the DM cohorts in the present study. We did not review the choroidal vasculature in the present study mainly because we did not have good axial measurements and did not control for factors that may affect choroidal volume variability.

Several studies have quantified the retinal thickness in different retina layers, which helps understand the pathologic processes that occur in DM and DR^18–29^. The present study quantified retinal thickness based on two divisions of the neurosensory retina with its vascular landmarks. The neuroretinal and vascular remodeling of the retinal architecture could be explained by molecular processes, such as matrix metalloproteinases, which can cause degradation of extracellular matrices, inflammation, and apoptosis of retinal capillaries in the context of diabetes and diabetic retinopathy^30^. We believe that it would be important to utilize multi-modal imaging, including OCTA, in order to capture these subtle changes in the retina. However, the implications of these subtle changes may be important and crucial in helping manage DR, as we have already found that changes may result in reduced vision. As DM remains a significant economic and healthcare burden^31^, all healthcare professionals should make continuous efforts to diagnose and treat it early.

There are several limitations in the present study. Although the two cohorts of DM patients are not significantly different in terms of the different severities of DR, it would be better to represent all DR severities, which would require a larger sample size. Secondly, this was a cross-sectional study. A longitudinal study of how the retinal thickness measurements change over time would shed light on the pathophysiologic sequence of events leading to macular thinning in DM. Although not routinely done in clinical practice, obtaining axial length may serve as an important data confounder, and should be performed in cases where cross-sectional retinal thickness measurements are compared. Lastly, to utilize retinal imaging and correlation with specific diseases, it would be ideal to include the foveal avascular zone in subsequent studies to correlate the cross-sectional and vascular findings.

In conclusion, patients with diabetes mellitus exhibit structural alterations in the macula. Patients with PRVW exhibit lower retinal thickness measurements in the inner and outer retina. The findings from this study support our previous finding of decreased visual acuity among patients with peripheral retinal vessel whitening that may be caused by loss of retinal tissue. Additionally, the findings in the present study provide further support for the use of PRVW as a prognostic indicator in DR.

## Data Availability

All de-identified data in the current study are available from the corresponding author (SB) upon reasonable request.
